# Sleep health of adults with cerebral palsy: A systematic review

**DOI:** 10.1111/dmcn.70330

**Published:** 2026-06-08

**Authors:** Deepika Nagabhushan, Caleb Kim, Daniel G. Whitney, Rita Ayyangar, Angeline Bowman, Sonia Sharma, Maxwell S. Barnish, Daniel Whibley

**Affiliations:** ^1^ College of Medicine Lake Erie College of Osteopathic Medicine Erie PA USA; ^2^ School of Medicine University of Washington School of Medicine Seattle WA USA; ^3^ Department of Orthopaedic Surgery University of Michigan Ann Arbor MI USA; ^4^ Department of Physical Medicine and Rehabilitation University of Michigan Ann Arbor MI USA; ^5^ Department of Medicine University at Buffalo Buffalo NY USA; ^6^ Peninsula Technology Assessment Group (PenTAG), School of Public Health and Sport Sciences University of Exeter Exeter UK

## Abstract

**Aim:**

To assess the evidence on the sleep health of adults with cerebral palsy (CP).

**Method:**

A comprehensive search of Ovid MEDLINE, Embase, and EBSCO CINAHL was conducted up to February 2025. Studies providing data on sleep‐related measures among adults with CP were included. Data were extracted on study design, demographics, sleep assessment methods, and sleep‐related findings. Risk of bias was assessed using a tool designed for prevalence studies.

**Results:**

From 2778 search records, 13 studies met inclusion criteria. Studies were published between 2015 and 2025 and conducted in the USA (*n* = 3), Europe (*n* = 7), Republic of Korea (*n* = 2), and Brazil (*n* = 1). Measures of sleep, sleep disorders, and sleep disturbances included clinical diagnoses, polysomnographic readings, and self‐reported sleep questionnaires. Findings suggest sleep‐related problems may be common, with some evidence pointing to higher rates than in adults without CP. However, most results were inconclusive due to small sample sizes and methodological variability.

**Interpretation:**

This review highlights a significant evidence gap with respect to the sleep health of adults with CP. This emphasizes the need for targeted research to generate generalizable data, with the potential to inform policy and clinical interventions to enhance sleep health and related quality of life of adults with CP.

## Graphical abstract

Although many people with cerebral palsy (CP) are adults, most sleep research in CP focuses on children. As poor sleep can affect pain, mood, energy, daily functioning, and quality of life we reviewed what is currently known about the sleep health of adults with CP. We searched three major research databases (MEDLINE, Embase, and CINAHL) up to February 2025 for studies that reported any sleep‐related measure in adults (18+) with CP. We summarized results across studies and assessed study quality. Thirteen studies met the criteria. Studies measured sleep in many different ways including clinical diagnoses, diagnostic codes in healthcare records, and self‐ or caregiver‐reported questionnaires. Overall, the evidence suggests that sleep problems are common for adults with CP and may be more frequent than in adults without CP. However, most findings could not be combined or generalized with confidence because studies were small, used different definitions and tools, and often did not include samples that represent the broader adult CP population. Important differences (for example by motor function level, intellectual disability status, or age) were rarely reported, limiting what we can say about who is most affected and why. This review highlights a need for better, more inclusive research that reflects the diversity of adults with CP and tracks sleep across adulthood so that care recommendations and services can be improved.

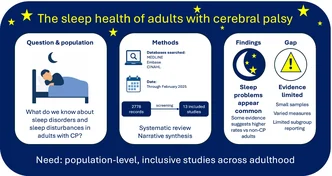

**Abbreviations**
ESS: Epworth Sleepiness Scale
OSA: obstructive sleep apnea
PROMIS: Patient‐Reported Outcomes Measurement Information System
PSQI: Pittsburgh Sleep Quality Index
SWAL‐QOL: Swallowing Quality of Life Questionnaire

**What this paper adds**

- There is limited research on sleep problems among adults with cerebral palsy (CP), such as obstructive sleep apnea and insomnia disorder.
- There is some evidence that adults with CP experience more sleep‐related problems than adults without CP.
- There is a need for well‐designed epidemiological studies of sleep in adults with CP.
- There is a need for research on the trajectory of sleep issues during transition to adulthood.

Cerebral palsy (CP) is a heterogeneous group of non‐progressive movement disorders that appears in early childhood and can significantly impact an individual's overall health and quality of life.^1^ Although a leading cause of childhood disability,^2^ most people living with CP in the USA are of adult age.^3^ It is therefore important to recognize and appreciate CP as a lifelong condition with neurological origins.^4^, ^5^, ^6^ As with the general population,^7^ sleep plays a vital role in supporting the physical, cognitive, psychological, emotional, and social health of adults with CP. Conversely, insufficient sleep is associated with higher levels of diabetes,^8^ hypertension,^9^, ^10^ heart disease,^11^ and stroke,^12^ all estimated to be higher among adults with CP than among their non‐CP counterparts.^13^ Sleep deficiency can also impact negatively on physical functioning,^14^ musculoskeletal pain,^15^ fatigue,^16^ and emotional well‐being.^17^ In short, sleep health is an important aspect of overall health and health‐related outcomes for adults with CP.

It is important to recognize the multidimensional nature of sleep. This is reflected in the SATED model that recognizes five important facets: satisfaction with sleep, alertness during waking hours, timing of sleep, sleep efficiency, and sleep duration,^18^ extended to the RU‐SATED model with the addition of sleep regularity.^19^ Meanwhile, Perrault et al.^20^ identified five sleep‐biopsychosocial profiles with distinct neural signatures. These profiles linked sleep variability to patterns of health, cognition, and lifestyle. Latent components described associations between poor sleep and psychopathology; sleep resilience and psychopathology; sleep aids use and sociability; sleep duration and cognition; and sleep disturbance, cognition, and psychopathology. Furthermore, Kolekar et al.^21^ posit biological factors (such as genetic predispositions, inflammatory markers [e.g. interleukin‐6, IL‐6; tumor necrosis factor‐alpha, TNF‐α], and circadian misalignment, and age‐related changes in melatonin), psychological factors (such as anxiety, rumination, perfectionism, and maladaptive sleep‐related cognitions), and social and environmental factors (such as shift work, digital device overuse, caregiving responsibilities, and socioeconomic stressors) as underlying insomnia. In addition, the sleep health of adults with CP intersects with multiple clinical concerns. Respiratory issues, orthopedic deformities, pain, and the use of supportive devices may contribute to further fragmented sleep and reduced satisfaction.^22^, ^23^, ^24^ Concomitant conditions, including epilepsy,^25^, ^26^ cognitive impairments,^27^, ^28^ and urinary incontinence,^29^, ^30^ may further compromise both the objective and subjective sleep experience. These interconnected physiological, psychological, and social dimensions relevant to adults with CP emphasize the need for reliable data on sleep health in this population.

A growing body of research is focused on estimating the extent and impact of sleep disorders and sleep disturbances among children with CP, with the most recent systematic review and meta‐analysis published in 2025.^31^ Incorporating new evidence into findings from a previous review published 6 years earlier,^32^ an estimated 25% to 50% of children with CP have a sleep disorder. These estimates are higher than those for children without CP^33^, ^34^ and reflect findings from individual studies that have shown that children with CP exhibit elevated rates of sleep‐disordered breathing^35^ and periodic limb movements during sleep^36^ compared to children without CP. This evolving evidence base highlights the importance of routine, comprehensive sleep assessment for children with CP, early intervention, provision of multidisciplinary care to address medical, environmental, and CP‐specific sources of sleep problems, and development of educational resources for caregivers.

While results from studies of the sleep of children with CP may be extrapolated to adults with CP, the validity of such generalization is open to question. It is possible that if sleep disorders or disturbances are identified and adequately treated in childhood, they will not sustain into adulthood. However, it is also possible that age‐related changes specific to both CP^37^ and/or sleep physiology^38^ may attenuate the clinical meaningfulness of any generalizations from the pediatric literature. Indeed, many of the physical, neurological, and psychological factors implicated in sleep disorders and disturbances among children with CP continue into adulthood (e.g. pain, mobility limitations, gastro‐esophageal reflux disease, respiratory issues, epilepsy, anxiety, depression, side‐effects of medications).^3^, ^39^, ^40^ For the adult population with CP, in the absence of robust evidence delineating the extent, severity, or individual impact of sleep problems on health‐related outcomes and quality of life, clinical and policy changes will remain, at best, a low priority and, at worst, completely neglected. Furthermore, adults with CP have difficulty finding guidance on sleep problems and are unaware that help is available and where to find it.^41^

Quantifying the extent and severity of sleep disorders and sleep disturbances among adults with CP is essential to help describe the burden and, in response, shape healthcare resource prioritization and allocation. Accordingly, we aimed to systematically review the literature to identify and, where possible and appropriate, synthesize data from studies that have assessed the sleep health of adults with CP, including occurrence of specific sleep disorders, sleep disturbances, and indicators of sleep quality. Our overarching goal was to provide an overview of the current evidence and identify gaps in need of further explication.

## METHOD

### Search strategy

This systematic review was conducted in line with the Cochrane Handbook^42^ and is reported in accordance with Preferred Reporting Items for Systematic Reviews (PRISMA) guidelines.^43^ A protocol for the review was prospectively registered on the PROSPERO database (CRD42023460621). Three electronic databases (Ovid MEDLINE, Embase, and EBSCO CINAHL) were systematically searched from inception to 22nd February 2025. Search strategies were developed with guidance from a university information specialist to identify studies of adults with CP that included information on the extent (e.g. prevalence) and/or severity of sleep disorders or disturbances or measures of sleep‐related parameters (search strategies provided in Table 1). Searches were not restricted by publication date or language. Results of the search were compiled in RefWorks (Clarivate, London, UK) software where duplicate records were removed. Lists were then exported and compiled in Microsoft Excel and manually screened to eliminate any remaining duplicates.

**TABLE 1:** Database search strategies.

Ovid MEDLINE	“‘cerebral pals*”.mp OR exp cerebral palsy/ AND sleep.mp OR apnea.mp OR apnoea.mp OR obstructive sleep apnea.mp OR OSA.mp OR insomnia.mp OR hypersomnia.mp OR “sleep‐dis*”.mp OR narcolepsy.mp OR “sleep dis*”.mp OR “brux*”.mp OR sleep quality.mp OR sleep quantity.mp OR sleep duration.mp OR circadian rhythm.mp OR exp sleep wake disorders/ OR exp sleep disorders, circadian rhythm/ OR restless leg.mp OR RLS.mp OR “parasomn*”.mp OR sleepiness*.mp’
Embase	“‘cerebral pals*”.mp OR exp cerebral palsy/ AND sleep.mp OR apnea.mp OR apnoea.mp OR obstructive sleep apnea.mp OR OSA.mp OR insomnia.mp OR hypersomnia.mp OR “sleep‐dis*”.mp OR narcolepsy.mp OR “sleep dis*”.mp OR “brux*”.mp OR sleep quality.mp OR sleep quantity.mp OR sleep duration.mp OR circadian rhythm.mp OR exp sleep wake disorders/ OR exp sleep disorders, circadian rhythm/ OR restless leg.mp OR RLS.mp OR “parasomn*”.mp OR sleepiness*.mp’
EBSCO CINAHL	‘cerebral pals* OR MM “cerebral palsy” AND sleep OR apnea OR apnoea OR OSA OR obstructive sleep apnea OR insomnia OR hypersomnia OR narcolepsy OR sleep‐dis* OR sleep dis* OR brux* OR sleep quality OR sleep quantity OR sleep duration OR circadian rhythm OR MM “Sleep Wake Disorders” OR MM “Sleep Disorders, Circadian Rhythm” OR RLS OR restless leg OR parasomn* OR sleepiness*’

### Study selection

Two reviewers (DN and DGW) independently screened titles and abstracts of all articles against eligibility criteria. In cases of disagreement, articles were progressed to a full‐article screening stage. At this stage the same reviewers (DN and DGW) independently assessed article content against eligibility criteria. If there was disagreement about eligibility, a third reviewer (DW) was consulted and the decision discussed among the team until consensus was reached. Reference lists of eligible studies were screened to identify any potentially relevant studies not detected by the search.

### Inclusion criteria

The inclusion criteria were structured according to the PICOS framework. Studies not meeting the inclusion criteria were excluded. The inclusion criteria were as follows. Population: adults (aged 18 years and above) with CP. Intervention: the aim of this review is to synthesize data on the prevalence and/or severity or symptom burden of sleep disorders and/or sleep disturbances among adults with CP. We operationalize sleep disorders as clinically diagnosed conditions and sleep disturbances more broadly as reported sleep‐related disruptions and/or symptoms.^44^, ^45^ Examples of sleep disorders include obstructive sleep apnea (OSA) and chronic insomnia disorder; examples of sleep disturbances include perception of poor sleep quality and non‐restorative sleep. Studies of trials of interventions were only considered eligible if baseline data on sleep disorders or sleep disturbances among a sample of adults with CP were provided. The purpose of only including baseline data from trials of interventions was to capture baseline occurrence/burden of sleep disorders or disturbances in selected samples without experimental intervention. Comparator: not applicable. Outcome: sleep disorders, sleep disturbances, or sleep‐related parameters. There were no additional outcomes. Study design: any quantitative study design that provided data on a sample of adults with CP and an estimated prevalence, frequency, or summary statistic related to a sleep disorder and/or sleep disturbance.

Only original full‐text articles published in peer‐reviewed journals were included; conference abstracts, editorials, reviews, and gray literature were excluded. If studies included data on both adults and children, data from the adult subgroup were extracted. In cases where separate data for adults and children were not provided, authors were contacted for adult subgroup data, and the study could only be included if these data were provided. The same approach was taken for mixed diagnosis studies; namely, where data were not presented separately for CP and other diagnoses, authors were contacted for CP subgroup data and the study only included if these data were provided.

### Data extraction and synthesis

Information was extracted on the following measures using a standardized form: study authors; year of publication; country of study conduct; study design; sampling frame; study inclusion and exclusion criteria; demographic characteristics (mean age, distribution of sex, CP type as reported); method of sleep assessment; sleep‐related results (prevalence, frequency or summary statistic related to a sleep disorder, sleep disturbance, and/or other sleep‐related parameter).

Data extraction was conducted by two independent reviewers (DN and SS) and cross‐checked by a third (DW). The items were open‐ended rather than close‐coded, but reviewers were asked to be internally consistent in their terminology where possible. The form was piloted before roll‐out for full use, and no modifications were made after the piloting process had been completed.

In the first instance, findings from eligible studies were narratively synthesized. Quantitative meta‐analyses were planned when at least two studies reported the same sleep outcome and were sufficiently comparable to justify pooling, considering the following: compatible outcome definitions and measurement (e.g. same instrument and threshold, or comparable diagnostic code groupings) and a comparable reference period; comparable study design and sampling frame; and extractable data (e.g. numerator/denominator or prevalence with standard error/95% confidence interval). Risk of bias was also considered when deciding whether pooling would be appropriate. We did not use a fixed *I* ^2^ threshold to determine suitability for meta‐analysis as *I* ^2^ is unreliable when the number of studies is small.^46^

### Risk of bias assessment

Risk of bias assessment of included studies was conducted by two independent reviewers (DW and AB) using the tool of Hoy et al.,^47^ which is designed specifically for the evaluation of prevalence studies. In epidemiological reviews of prevalence, it is important to use a tool that is specifically designed for prevalence studies because their nature is different from studies of interventions and therefore a risk of bias tool designed for studies of interventions will not identify the most important limitations in studies of prevalence. Although studies did not necessarily have to be population‐level studies of prevalence, this tool was used to help highlight areas for methodological attention in future high‐quality prevalence studies of sleep disorders and disturbances among adults with CP and to evaluate the extent to which the study could provide a valid estimate for adults with CP. The assessment tool is composed of 10 items, four of which evaluate external validity and six evaluate internal validity. A summary judgement of risk of bias is determined on balanced consideration of assessment of all 10 items, resulting in a categorization of low (further research very unlikely to change confidence in the estimate), moderate (further research likely to have an important impact on confidence in the estimate), or high (further research likely to change the estimate) risk of bias. This approach reflects recommendations from Cochrane and Grading of Recommendations Assessment, Development and Evaluation (GRADE).^42^, ^48^

## RESULTS

### Search results

Of 2778 records retrieved by the search, 91 full texts were assessed for eligibility and 13 met the inclusion criteria (Figure S1). Nine of the screened texts included data on sleep from samples comprising both children and adults. On emailing corresponding authors of these papers, two provided the data requested^49^, ^50^ and are included in the findings described below. No potentially eligible mixed diagnosis studies were identified where separate assessment for people with CP was possible.

### Study profile

Eligible studies were published between 2015 and 2025 and conducted in the USA (*n* = 3),^51^, ^52^, ^53^ the Netherlands (*n* = 3),^54^, ^55^, ^56^ Brazil (*n* = 1),^57^ Portugal (*n* = 1),^58^ Spain (*n* = 1),^49^ Republic of Korea (*n* = 2),^59^, ^60^ and Sweden (*n* = 2).^50^, ^61^ Eight were cross‐sectional observational studies,^49^, ^51^, ^53^, ^54^, ^56^, ^58^, ^59^, ^61^ two reported baseline data from clinical trials,^57^, ^60^ one reported baseline data from a longitudinal cohort study,^52^ one was a case–control study,^50^ and one used a micro‐longitudinal design.^55^ Five studies used a registry or other large pre‐existing dataset as the sampling frame;^50^, ^51^, ^52^, ^53^, ^61^ the remaining eight recruited participants through clinical or community sources (in one case explicitly using convenience sampling^58^). Across the included evidence base, sleep outcomes were operationalized in three distinct ways, which informed interpretation throughout the synthesis: (1) diagnosed sleep disorders (five studies) captured by polysomnography,^57^ administrative diagnostic codes,^50^, ^51^, ^52^ or clinical guideline‐based classification;^58^ (2) self‐ or proxy‐reported sleep symptoms/disturbance measured using questionnaires or study‐specific items (*n* = 6 studies);^49^, ^53^, ^54^, ^55^, ^56^, ^61^ and (3) sleep‐related quality‐of‐life impact captured as a subdomain of condition‐specific quality‐of‐life instruments (*n* = 2 studies).^59^, ^60^ Reporting of key subgroups (e.g. Gross Motor Function Classification System [GMFCS] level, intellectual disability, age strata) was inconsistent; subgroup‐specific synthesis was therefore only possible in a limited number of instances, which we note where available. Two studies reported findings stratified by GMFCS level,^54^, ^61^ and one study restricted inclusion to participants without intellectual disability.^54^ Two studies focused on narrow age bands (20–22 years; 21–34 years).^54^, ^61^ A summary of included studies and their sleep‐related findings is shown in Table S1.

### Specific sleep disorders

Five studies provided data on specific sleep disorders among adults with CP; one assessed OSA,^57^ one assessed sleep bruxism,^58^ and three reported estimates for grouped sleep disorders using diagnostic codes.^50^, ^51^, ^52^ In the sections below we present findings by disorder type and measurement approach, and interpret results in the context of study design and sampling frame (e.g. objective physiology in small clinical samples versus diagnostic coding in large administrative datasets).

#### Obstructive sleep apnea

Only one study assessed OSA.^57^ This study reported baseline data from a small clinical trial in Brazil (*n* = 13), recruiting from a single clinic and restricted to adults with ‘spastic diparetic CP’. The enrolled sample was relatively young (mean age 28 years). Sleep was assessed objectively using overnight polysomnography. Six participants (46%) met criteria for OSA (four mild, two moderate). Additional baseline polysomnographic parameters are reported in Table S1. No other subgroup stratifications were available.

#### Sleep bruxism

Only one study assessed sleep bruxism (excessive teeth grinding during sleep).^58^ This cross‐sectional study recruited a convenience sample from six institutions in Portugal (*n* = 110, mean age 42.9 years). Sleep bruxism was classified using American Academy of Sleep Medicine guidance and parental/legal guardian report when necessary. The prevalence of sleep bruxism was 14.5%. No additional stratified results were reported.

#### Grouped specific sleep disorders

Three studies assessed a grouped ‘any sleep disorder’ outcome using diagnostic codes in large healthcare datasets (sample sizes 5888 to 17 212).^50^, ^51^, ^52^ These studies provide estimates of diagnosed sleep disorders in healthcare systems, but do not differentiate specific disorders. One baseline analysis of a longitudinal US cohort using a large claims database and identifying sleep disorders using ICD‐9 diagnostic codes reported a sleep disorder prevalence of 4.5% among adults without pain, 6.1% among those with new pain, and 10.1% among those with consistent pain.^52^ One case–control study in Sweden using a population registry (adult subgroup data provided by the authors) identified sleep disorders using ICD‐10 diagnostic codes. The estimated probability of an adult with CP having a diagnosed sleep disorder was 0.65% (standard error 0.05), higher than matched controls, OR 1.8 (95% CI 1.5–2.2).^50^ One cross‐sectional study using US Medicare data and ICD‐10‐CM diagnostic codes reported a sleep disorder prevalence of 24.6% among adults with a mood affective disorder and 10.3% among those without a mood affective disorder.^51^ This study included adults of approximately working age, whereas the other two studies sampled a broad range of adult ages. No studies reported estimates stratified by GMFCS or intellectual disability status for code‐defined sleep disorders.

### Sleep disturbances/symptom burden (non‐diagnostic)

Eight studies reported non‐diagnostic sleep‐related outcomes using self‐report items or questionnaires including sleep quality, quantity and sleep medication use (*n* = 1 study),^61^ Patient‐Reported Outcomes Measurement Information System (PROMIS) sleep disturbance measures (*n* = 2 studies),^53^, ^54^ items from the Swallowing‐Quality of Life Questionnaire (SWAL‐QOL) (*n* = 2 studies),^59^, ^60^ the Pittsburgh Sleep Quality Index (PSQI) (*n* = 3 studies),^49^, ^55^, ^56^ and the Epworth Sleepiness Scale (ESS) (*n* = 1 study).^49^ These measures characterize symptom burden (and, in the case of SWAL‐QOL, sleep‐related quality‐of‐life impact) rather than clinically diagnosed sleep disorders.

#### Sleep quality, quantity, and use of sleep medications

One study assessed sleep quality, quantity, and use of sleep medications.^61^ This cross‐sectional study sampled young adults with CP (*n* = 61, age 20–22 years) from a local registry in Sweden. Sleep outcomes were self‐reported (CP diagnosis was registry‐based). Forty‐one per cent reported significant sleep problems (not further specified as quality vs quantity). This study provided GMFCS‐stratified results: sleep problems were most common in GMFCS level V (70%). Of the total sample, 17% reported using sleep medication at least weekly. There was no evidence to assess other stratification groupings.

#### PROMIS sleep disturbance (symptom severity; T‐scores)

Two cross‐sectional studies reported PROMIS sleep disturbance scores, which reflect perceived sleep problems, including difficulties with sleep initiation, maintenance, and overall quality (responses converted to a standardized T‐score to facilitate comparison to a reference population with a mean T‐score of 50 and standard deviation of 10, scores >50 indicating greater sleep disturbance).^53^, ^54^ Both were cross‐sectional observational studies, one^53^ conducted in a US setting, the other^54^ in the Netherlands. Gannotti et al.^53^ considered a broad population with CP, while Van Gorp et al.^54^ only considered those without intellectual disability. While the Dutch study^54^ recruited younger adults (*n* = 97, age 21–34 years), the US study^53^ had a mean age of 42 years (*n* = 263). It should be noted that in the Dutch study^54^ the authors computed a comparator T‐score for the Dutch general population but this had an older age profile than the study sample (mean 50.9 years, standard deviation [SD] 8.3 years). Van Gorp et al.^54^ used the Dutch‐Flemish translation of the PROMIS version 1.0 Sleep Disturbance Short Form 8a. Mean (SD) T‐score was 50.1 (7.8). This did not vary significantly by GMFCS level (Table S1) and was lower (indicating less sleep disturbance) than the comparator T‐score for the general Dutch population. In comparison, Gannotti et al.^53^ used four sleep‐related items from the PROMIS‐29, evaluating sleep quality, disturbance, effects on daytime functioning, and perception of being rested and refreshed upon awakening. Among 263 adults with CP (mean age 42 years), the median (interquartile range) sleep disturbance T‐score was 53 (48–58). There was no evidence to assess other stratification groupings.

#### Items from the Swallowing Quality of Life Questionnaire

Two studies assessed sleep disturbances using the SWAL‐QOL.^59^, ^60^ Yi et al.^59^ was a cross‐sectional study, while Kim et al.^60^ provided baseline data from a telehealth trial. The 44‐item SWAL‐QOL tool enables assessment of how swallowing difficulties impact on multiple domains of quality of life, including sleep.^62^ Sleep domain scores can be converted to a 0 to 100 scale, with 0 indicating the worst effect of swallowing difficulties on sleep‐related quality of life. Both studies were conducted in Republic of Korea using a broad CP sample. Yi et al.^59^ had the larger sample although age profiles were comparable (Yi et al.^59^ *n* = 117, age range 20–79 years, mean age 38.1 years [SD 12.4] plus 177 adults without CP; Kim et al.^60^ *n* = 16, mean age 43.4 years [SD 10.4]). Yi et al.^59^ found that those with CP had a statistically significantly lower mean sleep domain score compared with the non‐CP sample (mean 61.0, SD 24.3, compared with mean 91.5, SD 11.2; *p* < 0.001). In comparison, Kim et al.^60^ only presented raw scores from the sleep domain of the SWAL‐QOL (in‐person group: mean 5.42, SD 3.26; telehealth delivery group: mean 7.44, SD 3.65), thus preventing direct comparison against the findings from Yi et al.^59^ There was no evidence to assess other stratification groupings.

#### Pittsburgh Sleep Quality Index

Three studies assessed sleep disturbances using the PSQI.^49^, ^55^, ^56^ The PSQI is a 19‐item tool that can be used to assess sleep quality over the previous month.^63^ It comprises 19 items grouped into seven components: subjective sleep quality, sleep latency (time taken to fall asleep), sleep duration, habitual sleep efficiency (proportion of time spent in bed asleep), sleep disturbances, use of sleep medications, and daytime effects of sleep disturbance. A summary score ranges from 0 to 21, with higher scores indicating poorer sleep quality, and a score greater than 5 commonly used to indicate poor sleep quality. All three studies were conducted in European settings (Spain^49^ and the Netherlands^55^, ^56^). Two^49^, ^56^ were cross‐sectional, while Verschuren et al.^55^ used a microlongitudinal design. All three recruited a broad population with CP. The mean age of the sample in Verschuren et al.^55^ and Riquelme et al.^49^ was under 30 years, reflecting a young adult population, whereas the sample in Van Rijssen et al.^56^ was older (median age 42 years). In Verschuren et al.^55^ (*n* = 32), median (interquartile range) PSQI score was 8.5 (6.0–9.8), with 84% categorized as having significant difficulties with sleep. This compared with a mean (SD) score of 5.7 (6.6) in the study by Riquelme et al.^49^ (adult subgroup *n* = 44, data provided by the authors on request) and a median (range) of 8 (3–16) in the study by van Rijssen et al.^56^ (*n* = 110) in which 91% of the sample were categorized as having significant difficulties with sleep. There was no evidence to assess other stratification groupings.

#### Epworth Sleepiness Scale

One study reported ESS scores.^49^ ESS is a self‐report measure used to assess excess daytime sleepiness.^64^ It comprises eight items that ask respondents to rate their likelihood of dozing off or falling asleep in different common daily situations (e.g. watching television). Item responses range from 0 to 3, with a summary score range of 0 to 24 (higher score indicating higher level of sleepiness). A score greater than 10 is an indicator of excessive daytime sleepiness. In an adult subgroup (*n* = 44; data provided by Riquelme et al. on request), mean ESS score was 6.3 (SD 6.6). There was no evidence to assess other stratification groupings.

### Summary of synthesis

Across 13 studies, evidence on adult CP sleep is heterogeneous in both design and measurement. Diagnostic outcomes were provided by polysomnography in one small, selected clinical sample (OSA) and through administrative diagnostic codes in three large datasets (grouped sleep disorders). These sources address different constructs (objective physiology and healthcare‐coded diagnoses) and cannot be interpreted interchangeably. Non‐diagnostic outcomes were more commonly reported and primarily reflect self‐reported symptom burden (e.g. PROMIS, PSQI, ESS) or sleep‐related quality‐of‐life impact in the context of dysphagia (SWAL‐QOL). Key stratification variables (GMFCS, intellectual disability, and age bands) were very rarely reported, limiting subgroup inference. Overall, the available evidence suggests that sleep‐related problems are common among adults with CP and, where comparator data exist, may be more frequent than in adults without CP; however, the predominance of selected samples, heterogeneous measures, and limited population‐representative designs constrain clinically definitive conclusions and preclude meaningful quantitative pooling at present.

### Quality assessment

Of the 13 studies included in this review, 12 were judged to be at high risk of bias with respect to providing valid prevalence estimates. This is unsurprising given the quality assessment was undertaken from the perspective of providing valid population‐level estimates of sleep disorders or sleep disturbance among adults with CP and the paucity of studies that set out with this specific aim (Table 2). Studies tended to be rated at high risk of bias for items focused on external validity (reflecting the extent to which their findings represented the broader adult population with CP) and more favorably for internal validity (less within‐study systematic error). The only study that specifically aimed to generate population‐level estimates was judged to be of moderate bias due to the inclusion of proxy reports for data collection.^50^

**TABLE 2:** Quality assessment of included studies (ordered by year of publication).

	External validity				Internal validity						Summary
	Was the study's target population a close representation of the national population in relation to relevant variables, e.g. age, sex, occupation?	Was the sampling frame a true or close representation of the target population?	Was some form of random selection used to select the sample OR was a census undertaken?	Was the likelihood of non‐response bias minimal?	Were data collected directly from the participants (as opposed to a proxy)?	Was an acceptable case definition used in the study (i.e. a case definition of the sleep disorder or sleep disturbance that was measured)?	Was the study instrument that measured the parameter of interest (e.g. presence and/or extent of sleep disorder/disturbance) shown to have reliability and validity (if necessary)?	Was the same mode of data collection used for all participants?	Was the length of the shortest prevalence period for the sleep‐related parameter of interest appropriate?	Were the numerator(s) and denominator(s) for the sleep‐related parameter of interest appropriate?	
Giannasi et al.^57^	No	No	No	No	Yes	Yes	Yes	Yes	Yes	Yes	High risk of bias
	High risk	High risk	High risk	High risk	Low risk	Low risk	Low risk	Low risk	Low risk	Low risk	
Jacobson et al.^61^	No	Yes	Yes	No	No	No	No	Yes	No	Yes	High risk of bias
	High risk	Low risk	Low risk	High risk	High risk	High risk	High risk	Low risk	High risk	Low risk	
van Gorp et al.^54^	No	No	No	No	Yes	Yes	Yes	Yes	Yes	N/A	High risk of bias
	High risk	High risk	High risk	High risk	Low risk	Low risk	Low risk	Low risk	Low risk		
Yi et al.^59^	No	No	No	Uncertain	Yes	No	No	Yes	Uncertain	N/A	High risk of bias
	High risk	High risk	High risk		Low risk	High risk	High risk	Low risk			
Whitney et al.^51^	No	Yes	Yes	Yes	No	Yes	Yes	Yes	Yes	Yes	High risk of bias
	High risk	Low risk	Low risk	Low risk	High risk	Low risk	Low risk	Low risk	Low risk	Low risk	
Whitney et al.^52^	No	Yes	Yes	Yes	No	Yes	Yes	Yes	Yes	Yes	High risk of bias
	High risk	Low risk	Low risk	Low risk	High risk	Low risk	Low risk	Low risk	Low risk	Low risk	
Cabrita et al.^58^	No	No	No	Yes	No	Yes	No	No	Yes	Yes	High risk of bias
	High risk	High risk	High risk	Low risk	High risk	Low risk	High risk	High risk	Low risk	Low risk	
Riquelme et al.^49^	No	No	No	No	No	Yes	Yes	Yes	Uncertain	N/A	High risk of bias
	High risk	High risk	High risk	High risk	High risk	Low risk	Low risk	Low risk			
Verschuren et al.^55^	No	No	No	Uncertain	Yes	Yes	Yes	Yes	Yes	N/A	High risk of bias
	High risk	High risk	High risk		Low risk	Low risk	Low risk	Low risk	Low risk		
Giannotti et al.^53^	No	No	No	No	Yes	Yes	Yes	Yes	Yes	N/A	High risk of bias
	High risk	High risk	High risk	High risk	Low risk	Low risk	Low risk	Low risk	Low risk		
Linder et al.^50^	Yes	Yes	Yes	Yes	No	Yes	Yes	Yes	Yes	Yes	Moderate risk of bias
	Low risk	Low risk	Low risk	Low risk	High risk	Low risk	Low risk	Low risk	Low risk	Low risk	
Kim et al.^60^	No	No	No	No	Yes	No	No	Yes	Uncertain	N/A	High risk of bias
	High risk	High risk	High risk	High risk	Low risk	High risk	High risk	Low risk			
van Rijssen et al.^56^	No	No	No	No	Yes	Yes	Yes	Yes	Yes	N/A	High risk of bias
	High risk	High risk	High risk	High risk	Low risk	Low risk	Low risk	Low risk	Low risk		

## DISCUSSION

To our knowledge, this is the first review to compile information on sleep disorders or disturbances focused exclusively on adults with CP. Only 13 studies met our inclusion criteria and, overall, the quality of data from the perspective of robust epidemiological estimates is weak, revealing a significant evidence desert in need of focused attention. This provides a stark contrast to the richer evidence available for children with CP, which has been able to support multiple high‐quality reviews of sleep health.^31^, ^32^, ^65^, ^66^, ^67^ Most studies included in this review report mean ages around 35 to 40 years. Given the increasing recognition of CP as a lifelong condition, it is important to note this age range and that significant changes may occur after the age of 40 years that may substantially impact sleep health. Furthermore, there was limited evidence addressing the impact of motor function and intellectual disability, as well as a reliance on self‐report measures for many outcome domains.

Almost all studies identified by our search did not aim to provide population‐level evidence; sampling from a small range of centers was common, although some studies used databases or registries, which may provide broader coverage and reduce some forms of selection bias. Accordingly, our assessment of quality is not a critique of individual studies but reflects the broader lack of attention to sleep epidemiology among the adult population with CP. Given this context, as expected, the external validity of estimates presented in this review is limited and they should not be interpreted as representative of the general adult population with CP. However, the internal validity of included studies was generally assessed at a lower risk of bias. This emphasizes the main area for growth in this research area – design and execution of population‐level studies using rigorous sampling techniques to provide generalizable results. Such studies could inform public health policies, guide healthcare resource allocation, and potentially help shape adult CP‐specific sleep intervention strategies. For optimal impact, future epidemiological study designs should ensure adequate representation of different demographic (e.g. age, sex, ethnicity, socioeconomic status) and CP‐specific factors (e.g. different types of CP; motor/tone abnormalities, namely spastic, dystonic, athetotic, choreatic, hypotonic CP; broad and rich GMFCS level coverage).

Data from studies of children with CP have consistently highlighted a high prevalence of sleep disorders and disturbances, including insomnia and sleep‐disordered breathing.^31^ Our review exposes a substantial knowledge gap with respect to the adult population with CP. Despite this, several potentially important findings are suggested by our review. Adults with CP seem to experience significant sleep‐related problems, for example as reflected in the consistently very high proportion categorized as having significant sleep issues when using the PSQI.^49^, ^55^, ^56^ However, studies using PROMIS measures did not provide corroboration.^53^, ^54^ This inconsistency, along with the small number and size of studies conducted so far, precludes any generalizations at this stage.

Although we had expected this to be a research area in need of development, we were surprised by the remarkable scarcity of epidemiological studies, particularly with respect to the most common sleep disorders. We identified no studies solely focused on the prevalence of insomnia disorder in adults with CP, and only one small trial providing data on OSA.^57^ The authors of the latter were themselves unable to identify prevalence studies of OSA in adults with CP, which they describe as notable given changes in physical and cognitive functioning among children with CP and OSA as they age. The non‐CP literature provides some support for this supposition.^68^, ^69^ Nevertheless, three studies provided estimates for undifferentiated sleep disorders.^50^, ^51^, ^52^ Furthermore, the studies by Whitney et al. included in this review provide evidence to show that sleep disorders are more common among adults with CP and comorbid mood affective disorders and/or chronic pain.^51^, ^52^ This mirrors findings from other populations where sleep, mood, and somatic problems are observed to overlap.^70^, ^71^, ^72^ This emphasizes the need for further examination of overlapping sleep disorders/disturbances and other conditions among adults with CP given the high occurrence of psychological and pain conditions in this population.^73^, ^74^ The extent of comorbid insomnia and sleep apnea among adults with CP is also in demand of evaluation given associated functional limitations indicated in other neurological conditions.^75^

Some studies were not included in our review as they did not provide subgroup data on adult samples. However, their results are coherent with our findings. For example, a cross‐sectional study (*n* = 28) with participants of age range 16 to 78 years reported a mean (SD) PSQI score of 7.6 (3.7), with 75% of the sample categorized as having poor sleep quality, and 57% sleeping less than the recommended 7 hours per night.^76^ Our strict exclusion criterion related to age could be argued as a limitation of our review, as could our search of three databases (MEDLINE via OVID, EMBASE, and CINAHL) and omission of gray literature. However, we intentionally set out to identify high‐quality peer‐reviewed evidence focused specifically on adults, balancing comprehensiveness with feasibility, to describe the current research landscape and inform the focus of future investigations. Regarding the databases searched, they provide extensive coverage of the relevant scientific literature and were prespecified in our PROSPERO‐registered protocol. However, we undertook a supplementary search of APA PsycINFO (EBSCOhost) using a comparable time frame and search terms as a quality assurance step. No additional eligible studies were identified beyond those already included. A further limitation is that it was not possible to systematically assess for selective outcome reporting bias in included studies as observational studies typically do not have a pre‐registered protocol.

Studies included in our review used different tools to assess sleep, sleep disorders, and sleep disturbances, preventing meaningful meta‐analysis. However, any such quantitative evidence synthesis would, we argue, be premature given the current absence of rigorous sampling techniques to provide generalizable insights. Once adult estimates are established, an additional area for investigation could be the trajectory of sleep disorders and disturbances during the transition from childhood to adulthood in the population with CP. This exploration could be enriched by studies into how age‐related changes in sleep architecture and CP‐specific challenges intersect to impact on sleep health. Insights from this endeavor may help inform early intervention strategies during childhood with sustainable effects throughout the life course.

This review was prospectively conceived and registered as a systematic review because our objective was to answer a focused question: the prevalence and/or severity of sleep disorders and sleep disturbances among adults with CP, using explicit methods to minimize bias (systematic searching, duplicate screening/extraction, and critical appraisal). As outlined by Munn et al.,^77^ systematic reviews are indicated when the intent is to address a specific question and critically appraise and synthesize available evidence to inform practice and future research, whereas scoping reviews may be undertaken to map a broader field, clarify concepts, or examine how research is conducted and commonly do not require formal risk of bias assessment. In practice, the limited and methodologically heterogeneous evidence base for adult CP sleep meant that quantitative pooling was not appropriate and our synthesis is necessarily descriptive, with prominent emphasis on measurement approaches and evidence gaps. We view this as a key finding rather than a limitation of review type; the systematic methods applied here identify where the current literature is insufficient to support robust, generalizable estimates and therefore delineates priority targets for future population‐based studies. As higher‐quality and more comparable studies emerge, a future update of this systematic review will be better positioned to provide pooled prevalence estimates and more robust clinical and policy guidance.

In the broader sleep medicine literature, a move towards multidimensional sleep assessment is gaining traction^18^, ^78^, ^79^, ^80^ and could be meaningfully applied to adults with CP. This could incorporate subjective reports with objective measures from wearable or ‘nearable’ devices as has been initiated in studies of children with CP.^81^ However, the validity of ‘objective’ estimates from devices should be borne in mind. Indeed, development of a CP‐specific multidimensional sleep health assessment framework may be warranted. Certainly, more polysomnographic data on adults with CP would be illuminating. Pursuing these efforts in tandem would support a more nuanced understanding of the sleep health of adults with CP. Noting the distinction between sleep disorders and disturbances, it should be considered whether interventions and treatment should differ between these groupings, as, for example, sleep hygiene rules are generally not adequate to address disorders. Future research should explicitly link findings to biopsychosocial models of sleep, potentially using realist review methods, to better understand the mechanisms underlying changes in sleep disturbances and disorders in adults with CP.

## CONCLUSION

This systematic review exposes a critical evidence gap about the sleep health of adults with CP. The paucity of generalizable data calls for increased efforts to determine the prevalence and subsequently the consequences of sleep disorders and disturbances in this population. This will pave the way for more informed clinical and public health responses to support the long‐term well‐being of adults with CP.

## CONFLICT OF INTEREST STATEMENT

The authors have no conflicts of interest to declare.

## Supporting information


**Appendix S1:** Study selection and characteristics of included studies.

**Figure S1:** PRISMA flowchart.

**Table S1:** Included studies.

## Data Availability

Template data collection forms, data extracted from included studies, and any other materials used in the review can be requested from the corresponding author.
