# Supplementary material for: Sleep health of adults with cerebral palsy: A systematic review
Source: Dev Med Child Neurol. 2026 Jun 8;68(12):1645–56. doi: 10.1111/dmcn.70330 (PMC13743678; doi:10.1111/dmcn.70330)
Supplement: Supplementary file 1 [file DMCN-68-1645-s001.docx]

**Figure S1: PRISMA flowchart**

**Identification of studies via databases and registers**

2778 records identified from:

Databases (n =3)

MEDLINE = 574

Embase = 1902

CINAHL = 302

Records removed *before screening*:

**Total** number of duplicate records removed (n = 715)

Duplicates records removed in RefWorks (n = 249)

Duplicate records removed manually (n = 466)

**Identification**

Records excluded

(n = 1970)

Records screened

(n = 2063)

Reports not retrieved

(n = 2)

Reports sought for retrieval

(n = 93)

**Screening**

Reports excluded: 78

Duplicate (n = 1)

Outcomes (n = 12)

Population (n = 34)

Publication type (n = 12)

Study design (n = 19)

Reports assessed for eligibility

(n = 91)

Studies included in review

(n = 13)

Reports of included studies

(n = 13)

**Included**
