# Supplementary material for: Sleep health of adults with cerebral palsy: A systematic review
Source: Dev Med Child Neurol. 2026 Jun 8;68(12):1645–56. doi: 10.1111/dmcn.70330 (PMC13743678; doi:10.1111/dmcn.70330)
Supplement: Supplementary file 2 [file DMCN-68-1645-s002.docx]

**Table S1. Included studies (**N=13; ordered by year of publication**)**

| **Reference** | **Study design and sampling frame** | **Study eligibility criteria** | **Method of sleep assessment** | **Sample size and characteristics** | **Sleep-related results** |
| --- | --- | --- | --- | --- | --- |
| Giannasi et al. 2015^57^ | Baseline data from a single-arm clinical trial investigating effects of neuromuscular electrical stimulation therapy on masticatory muscles and sleep in adults with CP.  Patients attending the Special Care Needs Clinic of the State of Sao Paulo University. | Inclusion criteria:  Spastic diparetic CP.  At least partially preserved cognitive function.  Participant or legal guardian able to provide informed consent.  Exclusion criteria:  Orthodontic/orthopedic treatment of the jaws or therapies to reduce spasticity in the preceding six months. | Overnight polysomnography | N=13  54% male  Mean age: 28yrs (SD 2) | OSA positive: N=6 (46%)  N=4 mild OSA; 2 moderate OSA  Pre-intervention sleep variables (mean, SD):  Apnea Index: 6.3/hr (7.7)  Hypopnea Index: 1.0/hr (1.3)  AHI: 7.2/hr (7.0)  TST: 185.4min (97.3)  Sleep onset latency: 38.7min (42.0)  REM latency: 102.2min (61.6)  Arousal Index: 13.1 (11.7)  PLM Index: 2.9 (3.0)  N1: 27.0 (19.6)  N2: 37.1 (13.4)  N3: 22.3 (21.1)  REM: 4.4 (3.0)  Mean SaO_2_: 96.4% (1.8)  Minimum SaO_2_: 83.6% (3.0) |
| Jacobson et al. 2019^61^ | Cross-sectional study.  Local registry including all young adults diagnosed with CP living in the greater Stockholm area. | Inclusion criteria:  Diagnosed with International Classification of Diseases, 10^th^ revision code corresponding to CP | Sleep problems: sleep quality & quantity (problematic or not); sleep medications | N=61  56% male  Mean age: 21yrs 2mnths (SD 8mnths) | Sleep problems: N=24 (41%)  Taking sleep medication at least 1/wk: N=10 (17%)  Sleep problems by GMFCS-ER level:  I: N=8/25 (33%), missing data for N=1  II: N=3/8 (38%)  III: N=3/7 (43%)  IV: N=3/9 (33%)  V: N=7/12 (70%), missing data for N=2 |
| van Gorp et al. 2019^54^ | Cross-sectional study.  Adults with CP without intellectual disability who had previously participated in a pediatric rehabilitation research program in the Netherlands. | Inclusion criteria:  Age 21-34  Exclusion criteria:  Disorders in addition to CP affecting motor functioning  Intellectual disability (classified as having attended special education for children with intellectual disability) | PROMIS v1.0 Sleep Disturbance short form 8a (Dutch-Flemish translation) | N=97  61% male  Mean age: 28.5yrs (SD 3.8) | PROMIS Sleep Disturbance T-score mean (SD): 50.1 (7.8)  By GMFCS:  I: 48.1 (8.0)  II: 51.8 (8.0)  III-V: 52.8 (5.8)  Reference population (general Dutch population): 50.9 (8.3) |
| Yi et al. 2019^59^ | Cross-sectional study.  Adults with CP attending a specialized community center in South Korea. | Inclusion criteria:  Age ≥ 20 years  Confirmed diagnosis of CP  Exclusion criteria:  Unable to respond to >2 items of the Korean version of the Mini-Mental State Examination questionnaire | Two items from the Swallowing-Quality of Life Questionnaire (SWAL-QOL), 0-100 scale; 100 indicates least effect on QOL; 0 indicates the worst effect. | N=117  60% male  Mean age: 38.1yrs (SD 12.4) | SWAL QOL sleep item mean: 61.0 (SD 24.3)  (significantly lower than ‘healthy’ non-CP control group (mean 91.5 (SD 11.2), p<0.001) |
| Whitney et al. 2020a^51^ | Cross-sectional study.  US Medicare fee-for-service database | Inclusion:  Beneficiaries aged 18-64 with 12 months of continuous enrollment in a health plan in 2016 and at least 1 medical service utilization in 2016.  A single claim for any CP diagnosis (ICD-10-CM code)  Exclusion criteria:  Beneficiaries covered by the Health Maintenance Organization plans | Dichotomous sleep disorder variable with a positive categorization based on having an ICD-10-CM code for at least one of: insomnia, hypersomnia, circadian rhythm sleep disorder, sleep apnea, narcolepsy and cataplexy, parasomnia, sleep-related movement disorder, sleepwalking, sleep terrors, nightmare disorder, or other or unspecified sleep disorder. | N=17,212  Subgroup with no mood (affective) disorder: N=12,389  56% male  Mean age: 44.7yrs (SD 11.8)  Subgroup with mood (affective) disorder: N=4,823  47% male  Mean age: 46.7yrs (SD 11.4) | 10.3% of the subgroup with no mood (affective) disorder classified as having a sleep disorder.  24.6% of subgroup with mood (affective) disorder classified as having a sleep disorder. |
| Whitney et al. 2020b^52^ | Baseline data from a US longitudinal (retrospective cohort) study.  Data extracted from Optum Clinformatics® Data Mart database (2013–2016) | Inclusion criteria:  ≥18 years of age  CP diagnosis from at least one claim  At least one service utilization | Dichotomous sleep disorder variable with a positive categorization based on having an ICD-9 family or individual code for at least one of: insomnia, hypersomnia, circadian rhythm sleep disorder, sleep apnea, narcolepsy and cataplexy, parasomnia, sleep-related movement disorder, sleepwalking, sleep terrors, nightmare disorder, or other or unspecified sleep disorder, sleep disorders not due to a substance or known physiological condition. | N=5888  Subgroup without pain: N=3,726  55% male  Mean age: 44.8yrs (SD 19.3)  Subgroup with new pain: N=859  50% male  Mean age: 52.7yrs (SD 19.0)  Subgroup with consistent pain: N=1,303  50% male,  Mean age: 56.7yrs (SD 17.2) | Any sleep disorder:  4.5% of subgroup without pain  6.1% of subgroup with new pain  10.1% of subgroup with consistent pain |
| Cabrita et al. 2021^58^ | Cross-sectional study.  Convenience sample from six institutions in Portugal | Inclusion criteria:  People with CP who lived at or attended institutions for people with special needs. | 2001 American Academy of Sleep Medicine guidance for sleep bruxism diagnosis. | N=110  53% male  Mean age: 42.9yrs (SD 13.6) | Sleep bruxism: N=16 (14.5%) |
| Riquelme et al. 2023^49^ | Cross-sectional study.  Participants with CP identified by physiotherapists from specialized centers in Spain. | Inclusion criteria:  Diagnosis of cerebral palsy  Age > 6 years (data from sub-sample of adults ≥18yrs provided by authors) | Epworth Sleepiness Scale, Spanish version    Pittsburgh Sleep Quality Index, Spanish version | Data for adult sample (≥18yrs) provided by authors:  N=44  66% male  Mean age: 28.9yrs (SD 12.5) | Mean ESS 6.3 (SD 6.6)  Mean PSQI 5.7 (SD 6.6) |
| Verschuren et al. 2023^55^ | Micro-longitudinal study.  Participants recruited from two specialized rehabilitation centers in the Netherlands. | Inclusion criteria:  Ambulatory adults with CP with complaints of fatigue during daily activities and those with limited ability to perform intensive physical activity  Diagnosed with spastic CP according to the Surveillance of Cerebral Palsy in Europe  16 to 60 years of age (actual age range of the sample: 20-54)  Classified in GMFCS-ER levels I to III  No contraindication for maximal exercise  No severe communication or understanding problems that impede proper measurement performance  No history of botulinum neurotoxin A injections and/or serial casting in the past 3 months or surgery in the past 6 months | Pittsburgh Sleep Quality Index | N=32  34% male  Median age: 29.7yrs (IQR 24.6-40.3) | Median PSQI score (IQR): 8.5 (6.0-9.8)  ‘Poor sleep’ (PSQI >5): 84% |
| Gannotti et al. 2024^53^ | Cross-sectional study.  US CP community registry | Inclusion criteria:  Diagnosed with CP  >18 years of age  Ability to understand English  Ability to legally provide consent | PROMIS 29 sleep disturbance T-score (Four sleep disturbance items from the PROMIS-29) | N=263  21% male  Mean age: 42yrs (SD 14) | PROMIS 29 sleep disturbance T-score (median, IQR):  Full sample (N=263): 53 (48, 58)  Sub-sample reporting any functional decline (N=176): 54 (48, 60)  Subsample reporting no functional decline (N=87): 53 (46, 58) |
| Linder et al. 2024^50^ | Case-control study.  A register collated to include all persons living with CP in Sweden. | Inclusion criteria:  Having a diagnosis of CP and aged 5-65 between the years 2006-2015 and living in Sweden (data for adults aged >18 provided by authors).  Control group: Matched 5:1 on sex, municipality of residence and birth year from Swedish total population register. | Sleep disorders identified by ICD-10 codes (F51.0-F51.9, G47.0-G47.9) | CP group:  N=10,210  55.8% male  Mean age: 36.6 (SE 14.0)  Control (non-CP) group:  N=49,093  55.6% male  Mean age: 36.4 (SE 14.0) | Probability of being diagnosed with a sleep disorder was higher among persons with CP (0.65%, SE 0.05) compared to persons without CP (0.36%, SE 0.01).  OR of 1.8 (95% CI 1.5-2.2) (outcome: sleep disorder; ref group: non-CP). |
| Kim et al. 2025^60^ | Baseline data from a clinical trial comparing in-person to tele-practice-based speech-behavior therapy.  Patients with CP attending a hospital in South Korea with reduced speech intelligibility and difficulty swallowing. | Inclusion criteria:  Diagnosis of CP  Signs and symptoms of dysarthria  Signs and symptoms of dysphagia  Ability to fully understand and follow the treatment process.  Exclusion criteria:  COVID-19 infection | Swallowing-Quality of Life Questionnaire (SWAL-QOL) sleep items | N=16  56% male  Mean age:43.4 (SD 10.4) | Baseline SWAL-QOL sleep domain score:  In-person therapy group (N=7)  Mean: 5.42 (SD 3.26)  Telehealth therapy group (N=9)  Mean: 7.44 (SD 2.65) |
| van Rijssen et al. 2025^56^ | Cross-sectional study.  Adults with CP recruited via the online platform of the Dutch patient association for people with CP (CP Nederland). | Inclusion criteria:  ≥18 years of age with CP  Sufficient understanding of the Dutch language.  Able to independently complete study questionnaires. | Pittsburgh Sleep Quality Index, Dutch version | N=110  40% male  Median age: 42  Age range: 18-77 | Total sample (N=110):  Sleep duration, hrs (median, range): 7, 2-11  44% met sleep (at least 7 hours/night) recommendations; 32% reported sleeping less than 7 hours/night.  Sleep onset latency, mins (median, range): 20, 2-180  Use of sleep medication: 32%  PSQI global score (median, range): 8, 3-16  Poor sleepers (PSQI>5): 91% |

SD: Standard Deviation; CP: cerebral palsy; OSA: Obstructive Sleep Apnea; AHI: Apnea/hypopnea index; TST: Total Sleep Time; PLM: Periodic Limb Movement; REM: rapid eye movement; N1, N2, N3: sleep stages; SaO2: oxyhemoglobin saturation; GMFCS-ER: Gross Motor Function Classification System - Expanded & Revised; PROMIS: Patient-Reported Outcomes Measurement Information System; SE: Standard Error.
