# Supplementary material for: Sleep health of adults with cerebral palsy: A systematic review
Source: Dev Med Child Neurol. 2026 Jun 8;68(12):1645–56. doi: 10.1111/dmcn.70330 (PMC13743678; doi:10.1111/dmcn.70330)
Supplement: Supplementary file 3 [file DMCN-68-1645-s003.docx]

**Appendix**

List of included articles (n=13)

1. Cabrita JP, et al. Prevalence of bruxism in adults with cerebral palsy institutionalized in Lisbon. *Spec Care Dentist*. 2022;42:155-159.
2. Gannotti ME, et al. Adults with cerebral palsy and functional decline: A cross-sectional analysis of patient-reported outcomes from a novel North American registry. *Disabil Health J*. 2024;17:101593.
3. Giannasi LC, et al. Effects of neuromuscular electrical stimulation on the masticatory muscles and physiologic sleep variables in adults with cerebral palsy: a novel therapeutic approach. *PLoS One*. 2015;10:e0128959.
4. Jacobson DNO, et al. Health-related quality of life, pain, and fatigue in young adults with cerebral palsy. *Dev Med Child Neurol*. 2020;62:372-378.
5. Kim KM, et al. Efficacy of in-person and telepractice-based extended Lee Silverman Voice Treatment LOUD((R)) on dysarthria and dysphagia in adults with cerebral palsy. *Digit Health*. 2025;11:20552076251315296.
6. Linder A, et al. A life course perspective on mental disorders and psychopharmacologic drug use among persons living with cerebral palsy. *Eur J Paediatr Neurol*. 2024;53:144-54.
7. Riquelme I & Montoya P. Factors affecting the use of pain-coping strategies in individuals with cerebral palsy and individuals with typical development. *Children (Basel)*. 2023;10(1):131.
8. Van Gorp M, et al. Pain, fatigue, depressive symptoms and sleep disturbance in young adults with cerebral palsy. *Disabil Rehabil*. 2021;43:2164-71.
9. Van Rijssen IM, et al. The 24-hour physical activities in adults with cerebral palsy and their adherence to the 24-hour movement guideline. *Arch Phys Med Rehabil*. 2025;106:696-703.
10. Verschuren O, et al. Real-time daily fatigue, sleep, physical activity, and health-related fitness in adults with cerebral palsy. *Dev Med Child Neurol*. 2023;65:509-16.
11. Whitney DG, et al. Clinical factors associated with mood affective disorders among adults with cerebral palsy. *Neurol-Clin Pract* 2020;10:206-213.
12. Whitney DG, et al. Effect of pain on mood affective disorders in adults with cerebral palsy. *Dev Med Child Neurol*. 2020;62:926-932.
13. Yi YG, et al. Dysphagia-related quality of life in adults with cerebral palsy on full oral diet without enteral nutrition. *Dysphagia*. 2019;34:201-209.

List of articles excluded at full-text review (n=80, including unavailable articles)

*Duplicate (n=1)*

1. Giannasi LC, et al. Effects of neuromuscular electrical stimulation on the masticatory muscles and physiologic sleep variables in adults with cerebral palsy: a novel therapeutic approach. *PLoS One*. 2015;10:e0128959.

*Outcomes (n=12)*

1. Al Rajeh S, et al. A community survey of neurological disorders in Saudi Arabia: the Thugbah study. Neuroepidemiology 1993; 12(3): 164-78. No sleep outcomes.
2. Alriksson-Schmidt A, et al. Follow-up of individuals with cerebral palsy through the transition years and description of adult life: the Swedish experience. J Pediatr Rehabil Med 2014;7(1):53-61. No sleep outcomes.
3. Clanchy KM, et al. Evaluation of a physical activity intervention for adults with brain impairment: a controlled clinical trial. Neurorehabil Neural Repair 2016;30(9):854-65. No sleep outcomes.
4. Groff DG, et al. Influence of adapted sport on quality of life: perceptions of athletes with cerebral palsy. Disabil Rehabil 2009;31(4):318-26. No sleep outcomes.
5. Holmes C, et al. Home-based motorised cycling in non-ambulant adults with cerebral palsy: a feasibility study. Disabil Rehabil 2025;47(3):609-17. No relevant sleep prevalence measures (only measure is perceived impact on sleep post intervention).
6. Langerak NG, et al. Incidence of spinal abnormalities in patients with spastic diplegia 17 to 26 years after selective dorsal rhizotomy. Childs Nerv Syst 2009; 25(12):1593-1603. No sleep outcomes (sleeping item of the ODI is a measure of pain rather than sleep).
7. Lewin G, et al. Nature and prevalence of long-term conditions in people with intellectual disability: retrospective longitudinal population-based study. BMJ Open 2025;15(1):e090857. No sleep outcomes. Also mixed diagnosis.
8. McCarthy M, et al. Walking speed and patient-reported outcomes in young adults with cerebral palsy. 2022;64(10):1281-8. Data for sleep quality item PROMIS 57 not reported separately – requested from authors but not provided.
9. Margre ALM, et al. Caracterization [sic] of adults with cerebral palsy. Rev Bras Fisioter 2010;14(5):417-25. No sleep outcomes.
10. Rimmer JH, et al. Retrospective evaluation of a pilot eHealth/mHealth telewellness program for people with disabilities: Mindfulness, Exercise, and Nutrition To Optimize Resilience (MENTOR). Mhealth 2022;8:15. No relevant prevalence measure. Also mixed diagnosis (12 people with CP).
11. Robotham RJ, et al. Comparing self-report fatigue assessment tools for adults with cerebral palsy in a Danish context. Dev Neurorehabil 2024;27(1-2):34-43. No sleep measures (some sleep-related items on the fatigue scale, but these are measures of fatigue not sleep, and the results for these items are not presented separately).
12. Rodby-Bousquet E, et al. Prevalence of pain and interference with daily activities and sleep in adults with cerebral palsy. Dev Med Child Neurol 2021;63(1):60-7. No relevant prevalence measure. Only sleep VAR measure is ‘does pain interfere with sleep?’. Also age range (16-76 years) does not align with our criteria.

*Population (n=34)*

1. Arbour-Nicitopoulos KP, et al. A cross-sectional examination of the 24-hour movement behaviours in Canadian youth with physical and sensory disabilities. Disabil Health J;14(1):100980. Children and young people aged 12 to 21.
2. Bahrampour M, et al. Utility values for the CP-6D, a cerebral palsy-specific multi-attribute utility instrument, using a discrete choice experiment. Patient 2021;14(1):129-38. Participants were adults from the Australian general population.
3. Barabas G, et al. Care-load for children and young adults with severe cerebral palsy. Dev Med Child Neurol1992;34(11):979-84. Separable adult data requested but not provided.
4. Bensmail D, et al. Effect of intrathecal baclofen on sleep and respiratory function in patients with spasticity. Neurology 2006;67(8):1432-6. Mixed diagnosis – only one participant with CP. Insufficient sample size to analyse CP separately.
5. Braam W, et al. Melatonin treatment in individuals with intellectual disability and chronic insomnia: a randomized placebo-controlled study. J Intellect Disabil Res 2008;52(Pt 3):256-64. Mixed diagnosis – only 9 people with CP (adults and children). Insufficient sample size to analyse adults with CP separately.
6. Brunton LK, et al. Self-reported factors contributing to fatigue and its management in adolescents and adults with cerebral palsy. Disabil Rehabil 2021;43(7):929-935. Includes adults and children (age from 14 years). Not prioritised for requesting data specifically on participants aged 18 and above because sleep VAR is simply do you use sleep/rest to manage fatigue, which is a measure of fatigue not sleep.
7. Diot CM, et al. Robot-assisted gait training with Trexo Home: users, usage and initial impacts. Children (Basel); 2023;10(3):437. Children and young people (age 2 to 24). Unlikely to be sufficient data on adults to allow separate assessment.
8. Greydanus S, et al. Intellectual disability: Sleep disorders [Book chapter]. 2015. Children (also book chapter).
9. Hayashi M, et al. Multimodality evoked potentials in severe athetoid cerebral palsy: Correlation with clinical features and all-night polygraphical data. Brain Dev 1992; 14(3): 156-60. Age range 14 to 23 years. Total sample size 8. Insufficient to allow separate consideration of adults.
10. Hayashi M, et al. REM sleep abnormalities in severe athetoid cerebral palsy. Brain Dev 1990;12(5):494-7. Age range 15 to 30 years. Total sample size 10. Insufficient to allow separate consideration of adults. Also restricted to severe athetoid CP.
11. Husrevoglu S, et al. Evaluation of family impact of children with cerebral palsy and family caregiver's quality of life, sleep quality and individual physical education satisfaction: a mixed study. Turk J Physiother Rehabil 2024; 35(3): 342-51. Participants are caregivers. Also no estimate of sleep parameters for adults with CP.
12. Krach LE, et al. Satisfaction of individuals treated long-term with continuous infusion of intrathecal baclofen by implanted programmable pump. Pediatr Rehabil 2006;9(3):210-8. Age range 5 to 42 years. Also no relevant prevalence measure – therefore not prioritised for requesting data on participants aged 18 and above.
13. McCormack ZL, et al. Intrathecal versus oral baclofen: a matched cohort study of spasticity, pain, sleep, fatigue, and quality of life. PM R 2016;8(6):553-62. Mixed diagnosis – total of 32 participants across a wide range of spasticity types – insufficient sample size to consider people with CP separately.
14. McPhee PG, et al. A core outcome set for multimorbidity risk in individuals with cerebral palsy. Dev Med Child Neurol 2022;64(7):881-89. Separable adult data requested but not provided.
15. McPhee PG, et al. The formula for health and well-being in individuals with cerebral palsy: cross-sectional data on physical activity, sleep, and nutrition. Ann Rehabil Med. 2020;44(4):301–10. Separable adult data requested but not provided.
16. Mohon RT, et al. Sleep-related breathing disorders associated with intrathecal baclofen therapy to treat patients with cerebral palsy: A cohort study and discussion. NeuroRehabilitation 2021;48(4):481-91. Children.
17. Kitai Y, et al. A questionnaire survey on the efficacy of various treatments for dyskinetic cerebral palsy due to preterm bilirubin encephalopathy. Brain Dev 2020;42(4):322-28. Children.
18. Larsson SM, et al. Hip pain in adolescents with cerebral palsy: a population-based longitudinal study. Dev Med Child Neurol 2021;63(5):601-7.Children (aged 12-17).
19. Marquez-Vasquez JF, et al. Comparative efficacy of botulinum toxin in salivary glands vs. oromotor therapy in the management of sialorrhea in cerebral palsy impact on sleep quality. Front Rehabil Sci. 2022;3:875235. Children (aged 3 to 17).
20. Martinez de Zabarte Fernandez JM, et al. Caregiver burden in patients with moderate-severe cerebral palsy. The influence of nutritional status. An Pediatr (Engl Ed) 2021; 94: 311-7. Children (aged 4 to 15).
21. Melland HI, et al. Clinical evaluation of an automated turning bed. Orthop Nurs1999;18(4):65-70. Mixed diagnosis study – sample size insufficient for separate assessment of people with CP to be feasible (total sample size 24 split between degenerative disease, spinal cord injury, stroke, cerebral palsy, or back surgery).
22. Poindexter AR & Bihm EM. Incidence of short-sleep patterns in institutionalized individuals with profound mental retardation. Am J Ment Retard 1994; 98(6): 776-80. Separable adult CP data requested but not provided.
23. Santos JS, et al. Day/night melatonin content in cerebral palsy. Neurosci Lett 2018; 686:23-27. Children (aged 3-12).
24. Shiihara Y, et al. Monitoring arousal in severely multiply handicapped people by continuously recording skin conductance. Sleep Biol Rhythms 2016; 1: 165-6. Mixed diagnosis study (also adults and children). Total sample size 12. Insufficient to allow separate assessment of adults with CP.
25. Tan SS, et al. Long-term trajectories of health-related quality of life in individuals with cerebral palsy: a multicenter longitudinal study. Arch Phys Med Rehabil 2014;95(11):2029-39. Children and young people (age range 1 to 24 years). Given very rudimental sleep measure (a VAS), not prioritised for requesting data for only those participants aged 18 and above.
26. Theis N, et al. Leucine supplementation increases muscle strength and volume, reduces inflammation, and affects wellbeing in adults and adolescents with cerebral palsy. J Nutr 2021;151(1):59-64. Age range 12 to 25. Total sample size 21 – insufficient for separate consideration of those aged 18 and over. Also a very rudimentary measure of sleep.
27. Van den Broek N, et al. Obstructive sleep apnea in people with intellectual disabilities: adherence to and effect of CPAP. Sleep Breath2021;25(3):1257-1265. Mixed diagnosis – only 3 adults with CP. Sample size insufficient to allow separate consideration of adults with CP.
28. Voepel-Lewis T, et al. A high psychological and somatic symptom profile and family health factors predict new or persistent pain during early adolescence. Clin J Pain 2021;37(2):86-93. Children aged 9-12, also those with CP excluded.
29. Winfield DL, et al. Electroencephalography; sleep findings in cerebral palsy. Pediatrics 1955;16(1):88-92. Children.
30. Wood N & Brown S. An exploratory study: The effects of sleep systems on sleep quality, pain and carer goals for non-ambulant children and young people with cerebral palsy. J Rehabil Assist Technol Eng 2022;9:20556683211070729. Children (average age 11.5 years).
31. Yoshida K. Elastic retracted oral appliance to treat sleep apnea in mentally impaired patients and patients with neuromuscular disabilities. J Prosthet Dent 1999;81(2):196-201. Mixed diagnosis – total sample size n=5, not feasible to assess CP separately.
32. Gallien P, et al. [Pain in adults with cerebral palsy]. Ann Readapt Med Phys 2007;50(7):558-63.
33. Miyamoto A, et al. [Treatment with ramelteon for sleep disturbance in severely disabled children and young adults]. No To Hattatsu 2013;45(6):440-4.
34. Hori T & Kodama M. [Sleep patterns in cerebral palsied adult]. Shinrigaku Kenkyu 1974;44(6):289-95.

*Publication type (n=12)*

1. Brunton LK, et al. Contributing factors and self-management of fatigue in adolescents and adults with cerebral palsy. 2020. Conference abstract.
2. Eunson, et al. Hemiplegic cerebral palsy and epileptic status in sleep. 2006. Conference abstract.
3. Gallien, et al. Quality of life of adults with cerebral palsy living in Brittany. 2015. Conference abstract.
4. Gannotti M, et al. Adult cerebral palsy patient reported outcomes registry: preliminary report on changes across lifespan in gross motor, manual abilities, and communication function. 2020. Conference abstract.
5. Giannasi LC, et al. Sleep quality through overnight standard polysomnography in patients with cerebral palsy. 2014. Conference abstract.
6. Giannasi LC, et al. Sleep quality through overnight standard polysomnography in patients with cerebral palsy. 2015. Conference abstract.
7. Koerber KN, et al. Successful management of adult patients with cerebral palsy. <https://dimensionsofdentalhygiene.com/article/successful-management-adult-cerebral-palsy/>. Magazine article.
8. McPhee PG, et al. The formula for health and well-being: Cross-sectional data on physical activity, sleep, and nutrition in individuals with cerebral palsy. 2018. Conference abstract.
9. Rodby-Bousquet E, et al. Pain in adults with cerebral palsy. 2020. Conference abstract.
10. Van den Broek N, et al. Pain, fatigue, and sleep disturbances in young adults with cerebral palsy. 2019. Conference abstract.
11. Whitney DG, et al. Associations of pain, sleep disorders, fatigue, and comorbid neurodevelopmental conditions with depressive disorders among privately insured adults with cerebral palsy. 2019. Conference abstract.
12. Zhu, et al. A report on characteristics of cerebral palsy from Henan cerebral palsy register and rehabilitation management system. 2019. Conference abstract.

*Study design (n=19)*

1. Baxter P. Comorbidities of cerebral palsy need more emphasis--especially pain. Dev Med Child Neurol. 2013;55(5):396. Editorial.
2. Baylis M. 24-Hour postural care and use of sleep systems in cerebral palsy. Paediatr Child Health (Oxford) 2020; 30(8): 296-302. Review article.
3. Bradford Jones K, et al. Care of adults with intellectual and developmental disabilities: cerebral palsy. FP Essent 2015;439:26-30. Review article.
4. Collin, et al. Cerebral palsy: The adult perspective. 2000. Editorial.
5. Dan B. Sleep as therapy in neurodevelopmental disorders. Dev Med Child Neurol 2018;60(5):434. Editorial.
6. Didden R & Sigafoos J. A review of the nature and treatment of sleep disorders in individuals with developmental disabilities. Res Dev Disabil 2001; 22(4): 255-72. Review article.
7. Edgin J & Saletin JM. Sleep, brains, and behavior across ten neurodevelopmental disorders: Introduction to the special issue on sleep in developmental disabilities. Res Dev Disabil 2020;102:103636. Editorial.
8. Giannasi LC, et al. Effects of neuromuscular electrical stimulation, laser therapy and LED therapy on the masticatory system and the impact on sleep variables in cerebral palsy patients: a randomized, five arms clinical trial. BMC Musculoskelet Disord 2012;13:71. Study protocol.
9. Gringas P. Sleep disorders in cerebral palsy. Dev Med Child Neurol 2017;59(4):349-50. Editorial.
10. Hayward H, et al. Sleep disordered breathing in adults with cerebral palsy: what do we know so far? South Med J2021;114(6):339-42. Review article.
11. Humphreys G, et al. Sleep positioning systems for children and adults with a neurodisability: A systematic review. Br J Occup Ther 2019; 82(1): 5-14. Review article.
12. Jaseja H. Efficacy of vagal nerve stimulation in patients with cerebral palsy: emerging corroborative evidence. Clin Neurol Neurosurg 2011;113(7):603. Letter to the editor.
13. Kotagal S. Sleep in neurodevelopmental and neurodegenerative disorders. Semin Pediatr Neurol 2015;22(2):126-9. Editorial.
14. Morley A. Cerebral palsy and sleep disordered breathing. Breathe (Sheff) 2016;12(4):357-363. Review and case report of one child.
15. Pruitt DW, Tsai T. Common medical comorbidities associated with cerebral palsy. Phys Med Rehabil Clin N Am 2009;20(3):453-67. Review article (also focused on children).
16. Salinas R, Saguil A. Physical activity and the health of wheelchair users. Am Fam Physician 2022;105(5):534-537. Review article.
17. Smith SE, et al. Adults with cerebral palsy require ongoing neurologic care: a systematic review. Ann Neurol 2021;89(5):860-71. Review article.
18. Strobl WM, et al. The quality of sleep and digestion in cerebral palsy depends not only on the level of functional independence. Rev Assoc Med Bras (1992). 2024;70(5):e20231637. Letter to the editor.
19. Verschuren O, et al. The formula for health and well-being in individuals with cerebral palsy: physical activity, sleep, and nutrition. Dev Med Child Neurol 2016;58(9):989-90. Letter to the editor.

*Unavailable (n=2)*

1. Losasso, et al. Common oral findings in dental patients with cerebral palsy. 2014.. Article unable to be retrieved. Title indicates it is unlikely to be about sleep outcomes.
2. Sasaki, et al. Abnormal EEG and its neuropathology. Based on autopsy cases of severe cerebral palsy with idiocy [Japanese]. 1975. Article unable to be retrieved.
